# Towards the ionizing radiation induced bond dissociation mechanism in oxygen, water, guanine and DNA fragmentation: a density functional theory simulation

**DOI:** 10.1038/s41598-022-23727-3

**Published:** 2022-11-18

**Authors:** Santosh KC, Ramin Abolfath

**Affiliations:** 1 Chemical and Materials Engineering, San José State University, San José, CA 95192 USA; 2 Department of Radiation Physics, University of Texas MD Anderson Cancer Center, Houston, TX 75031 USA

**Keywords:** Cancer, Physics

## Abstract

The radiation-induced damages in bio-molecules are ubiquitous processes in radiotherapy and radio-biology, and critical to space projects. In this study, we present a precise quantification of the fragmentation mechanisms of deoxyribonucleic acid (DNA) and the molecules surrounding DNA such as oxygen and water under non-equilibrium conditions using the first-principle calculations based on density functional theory (DFT). Our results reveal the structural stability of DNA bases and backbone that withstand up to a combined threshold of charge and hydrogen abstraction owing to simultaneously direct and indirect ionization processes. We show the hydrogen contents of the molecules significantly control the stability in the presence of radiation. This study provides comprehensive information on the impact of the direct and indirect induced bond dissociations and DNA damage and introduces a systematic methodology for fine-tuning the input parameters necessary for the large-scale Monte Carlo simulations of radio-biological responses and mitigation of detrimental effects of ionizing radiation.

## Introduction

Deoxyribonucleic acid (DNA), is one of the key components of life which is responsible for the storage and transmission of genetic information^1,2^. It comprises a phosphate backbone and four nitrogen-containing bases, which are named adenine (A), cytosine (C), guanine (G), and thymine (T). It is found that A base pairs only with the T base, and the G base pairs with the C base^1,2^. DNA is an important part of life, but, it is very sensitive to the environment.

In radiotherapy, the interaction of mega-voltage ionizing radiation with biological systems causes ionization processes in biomolecules such as DNA, proteins, and their surrounding environment in cell nuclei. Among all of these ionization processes, DNA damage is critical to the clinical outcome of radiotherapy. After initial induction of DNA damage, a dynamical cascade of stochastic microscopic events and complex biochemical pathways, including, enzymatic homologous and non-homologous repair and misrepair end-joining determine the lethality of the irradiated cells.

Empirical studies in radio-biology and radio-chemistry have suggested induction of approximately 1000 single-strand breaks (SSBs) and 40 double-strand breaks (DSBs) per one gray (1 Gy = 1 J/kg ) of low linear energy transfer (LET) of ionizing radiation such as X- or $\gamma$-rays in typical mammalian cells^3–6^.

Accordingly, the level of DNA molecular base damage has been estimated to be around 2500–25,000 per gray in a cell. This is about 2.5–25 times the yield of sugar-phosphate induced damage in the DNA backbone.

On the other fronts, there is a tremendous concern about the risk of radiation in the human body while going into outer-space^7^. Outer-space consists of an ionizing radiation environment dominated by energetic and penetrating ions and nuclei. Thus, there is a risk of DNA damage due to ionizing radiation and a chance of radiation-induced cancer in manned space exploration^7,8^. Like in radiotherapy and radiobiology, there is a need for atomic-level understanding of biomolecules in radiation exposed in space. A large-scale computational model, relying on quantum dataset, will provide more realistic computational tools in assessing the biological risks due to space radiation, in particular for astronauts who are planning for the long-term exploration of other planets such as Mars. This is in alignment with NASA’s space radiobiology research that aims to mitigate the detrimental effects of the space radiation environment on the human body, a project focusing on the human presence outside of the relative protective Van Allen belt. Although the spacecraft itself somewhat reduces radiation exposure, it does not completely shield astronauts from galactic cosmic rays, which are highly energetic heavy ions, or from solar energetic particles, which primarily are energetic protons. By one NASA estimate, for each year that astronauts spend in deep space, about one-third of their DNA will be hit directly by heavy ions^9,10^ from Galactic Cosmic Radiation (GCR).

The occurrence of initial DNA damage has been classified into direct and indirect processes. In direct mechanism, ionization takes place mainly via direct electrodynamical coupling between the source of radiation and DNA molecule. Nuclear interaction with atomic nuclei is another possibility in causing direct DNA damage. For X- or $\gamma$-rays, depending on the energy of the incident photon, the coupling strength varies among photoelectric and Compton effects where shell electrons are ejected directly. In addition, high enough energy photons may interact with the nuclei of atoms and generate pair of electrons and positrons. Another source of uncharged particles such as neutrons may undergo nuclear interaction and make nuclear fragmentation and produce secondary charged particles as well as photons. The charged particles, either primary or secondary, interact with shell electrons through long-range Coulomb interaction. Under enough energy and momentum transfer, these charged particles eject shell electrons. Thus a “direct damage” originates from the direct ionization of a molecule, i.e., an isolated molecule (in vacuum) loses a number of shell electrons within atto-second (electromagnetic) time-scales and undergoes structural instability because of electrostatic charge imbalance and the repulsive forces among positively charged nuclei. The threshold of such instabilities requires a minimum number of ionizations and energy on a specific site of DNA.

In the indirect mechanism of radiation interactions, the radiation dominantly ionizes water molecules and creates neutral $^{\cdot }\hbox {OH}$ free radicals^3^. The DNA damage process involves the generation and diffusion of $^{\cdot }\hbox {OH}$ radicals in cell nuclei and/or aqueous environments followed by chemical reactions that allow the removal of hydrogen atoms from the DNA. This process is energetically favorable for $^{\cdot }\hbox {OH}$ radicals as it forms a water molecule and fills the electronic shell by neutralizing its magnetic moment.

We note that because of the aquatic environment in cells, approximately 70–80$\%$ of interactions take place through indirect damage and the rest are associated with the direct damage. In recent years, various types of molecular simulations were devoted to studying DNA damage by either free radicals, or direct damage^11–16^. This is in particular important to analysis of the recent experiments based on FLASH ultra high dose radiotherapy^16^.

Here we combine these two events to study their mutual effects. Moreover, many current computational platforms designed for the large-scale simulations of the DNA-damage at the nanoscopic scales^17–23^ lack accurate details from the first-principle direct and in-direct processes. For example the authors of Ref.^24^ quote direct ionization energies from fitting to the experimental data of DNA damage. Because of systematic uncertainties in calculating these fitted values, we aim to cover the gap in the details of the input parameters and allow the developers to update the tables used for MC simulation of DNA damage.

In this study, we focus on the simulation of combined direct and indirect damage to DNA molecules including base and backbone. As a representative of DNA-base, and without loss of generality, we focus on Guanine. We find as a combined function of ionization and hydrogen loss in indirect mechanism, the electrostatic repulsion of atomic nuclei dominates the electronic chemical bonds and is responsible for molecular fragmentation. Thus we quantify DNA fragmentation as a function of ionization and hydrogen abstraction. We show that at least four to five ionization must take place till the molecule undergo mechanical instability and fall apart. A molecule such as a DNA-base with nano-meter size extension, consists of several atoms and large number of electrons. Passage of a charged particle at low impact factors may result in a sequence of energy transfers to a single molecule that contains a large scattering cross-section. Thus, ejection of more than one shell electron from a single DNA nucleotide is possible if adequate energy transfers locally to a fragment of DNA by a high-energy photon or a charged particle.

The remainder of the paper is organized as follows. Section “Computational methods” introduces the calculation methods. The results and discussion are described in “Results and discussion”. “Conclusion” provides the conclusion of our study.

## Computational methods

First-principles calculations based on Density Functional Theory (DFT)^25,26^ were performed to investigate the charged defects in molecules such as H$_{2}$O, O$_{2}$, guanine, and DNA backbone, as there have been successful reports of using DFT-based computational approach with plane-wave basis set on Guanine and DNA-backbone^27,28^.

The core and valence electrons interactions were described within projector-augmented plane-wave (PAW) potentials as implemented in the Vienna Ab-initio Simulation Package (VASP)^29–31^. The exchange potential with the generalized gradient approximation of Perdew, Burke, and Ernzerhof (PBE)^32^ was included. Spin polarization and an energy cutoff of 500 eV were used for the plane-wave basis set in all the calculations. In the PAW potentials 2s$^{2}$ 2p$^{4}$, 3s$^{2}$ 3p$^{3}$, 2s$^{2}$ 2p$^{2}$, 1s$^{1}$ and 2s$^{2}$ 2p$^{3}$ electrons were explicitly treated as the valence electrons for O, P, C, H, and N, respectively.

First, the Oxygen and water molecules in a simulation box of size 15Å$\times$ 15Å$\times$ 15Å was optimized. Electrons were gradually removed from the system to observe the oxygen and water bond dissociation. Similarly, we have investigated the effect of electron extraction in Guanine and DNA sugar moiety of backbone. The charged molecules were relaxed until the Hellman–Feynman forces were less than 0.01 eV/Å.

**Figure 1:**
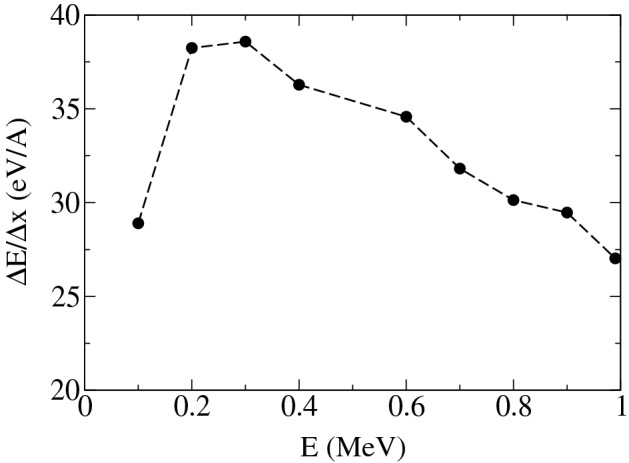
Linear energy transfer of a pencil beam of proton as a function of proton kinetic energy in water.

To address the question on possibilities whether a single molecule such as O$_2$, H$_2$O, guanine or a single DNA sugar-moiety could experience multi-ionization events and losing more than one electron, we provide an explanation based on scoring highly ionized track-structure of particles passing through these molecules, as given below. Figure 1 shows the result of event-by-event ionization, calculated by performing Monte Carlo simulation of a pencil beam of proton using Geant4-DNA^15,18^. The figure shows the linear energy deposition per length (LET) to create ionization events as a function of kinetic energy of proton in water. The energy transfer results in scattering and release of valence electrons and formation of secondary electrons. As shown, the energy density transferred to generate the secondary electrons from valence electrons within a length-scale of one Angstrom goes up to 40 eV for protons. For heavier charged particles such as alpha/He, Carbon, Fe, ... the particle LET goes up to orders of magnitude than the maximum LET of proton. Thus it is possible that a single molecule with linear spatial extension, from couple of Angstroms to 1 nm lose multiple electrons simultaneously.

**Figure 2:**
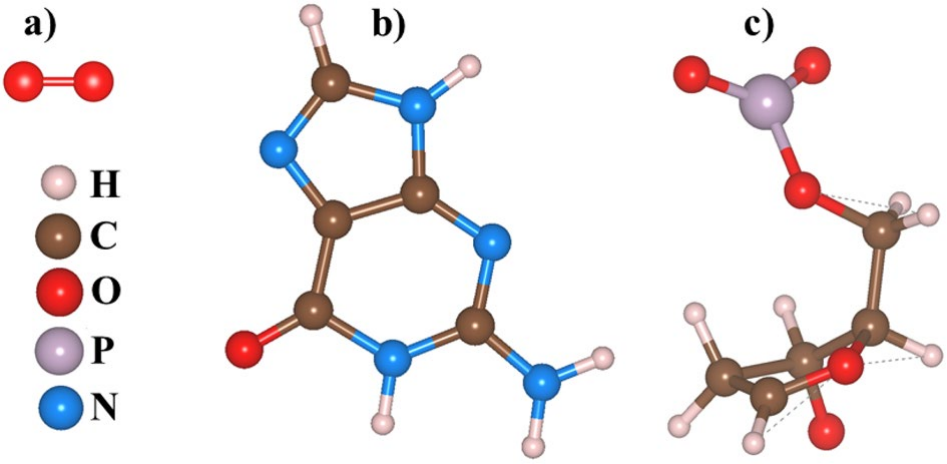
Optimized atomic structures of the Oxygen, Guanine (C$_{5}$H$_{5}$N$_{5}$O), and fragment of Deoxyribonucleic acid (DNA). (**a**) Oxygen, (**b**) Guanine and (**c**) a fragment of DNA.

## Results and discussions

Our DNA backbone model is similar to the deoxyribose residue used in other *ab-initio* calculations such as Ref.^33^ where the system of interest consists of a DNA nucleobase modeled by an amino group attached to the deoxyribose as shown in Fig. 2a–c. The deoxyribose sugar ring is an important component of nucleotides and plays a role in the stability of DNA double-helix structure. Any damage to the sugar ring causes breakage of strands and the formation of single-strand break (SSB). The formation of a pair of SSBs in opposite strands, within ten base pairs, leads to a single double-strand break (DSB).

Our DNA backbone model is similar to the deoxyribose residue used in other ab initio calculation such as Refs.^12,33^ where the system of interest consists of a DNA nucleobase modeled by an amino group attached to the deoxyribose in the presence of the OH-radical as shown in Figure 4 in Ref.^12^. In coarse-grained models, such as QM-MM, it is possible to add the entire structure of guanine (or any other base) to the sugar ring and expand the DNA fragments to a larger molecule, and treating a segment within QM and the other by classical molecular mechanics force field (MM), see for example Ref.^14^.

To study the effect of ionizing radiation on the molecules, we systematically performed the DFT calculation of molecules in various charge states. For a representative of DNA-base, we consider Guanine in addition to the oxygen and water molecules in our simulations.

### Effect of electron extraction on oxygen molecule

It is known that oxygen species play important roles in both tumor and normal cells under radiation. Typically, tumor cells contain less oxygen with a complex environment known as hypoxic, so they are more radio-resistant than normal tissues.

For low-linear energy transfer (LET) radiations, cells irradiated under normoxic conditions sustain about 2.9 times as many double-strand breaks (DSBs) as cells irradiated under anoxic conditions. This indicates that the greatly deficient in oxygen concentration has more impact compared to a normal oxygen concentration in the cell^34^.

It has also been suggested that oxygen depletion leads to lower normal-tissue toxicity at FLASH dose rates that take place within femto-to nanoseconds of irradiation where the biomolecular damage would be reduced in an environment with physoxic oxygen levels^16^. Also, there are specific transitions in oxygen that make the molecular oxygen toxic, such as singlet oxygen. Thus, it is important to understand the stability of oxygen molecule as it plays a crucial role in a so-called oxygen depletion effect and if the charge induced radiation environment facilitates the dissociation of oxygen molecule.

**Figure 3:**
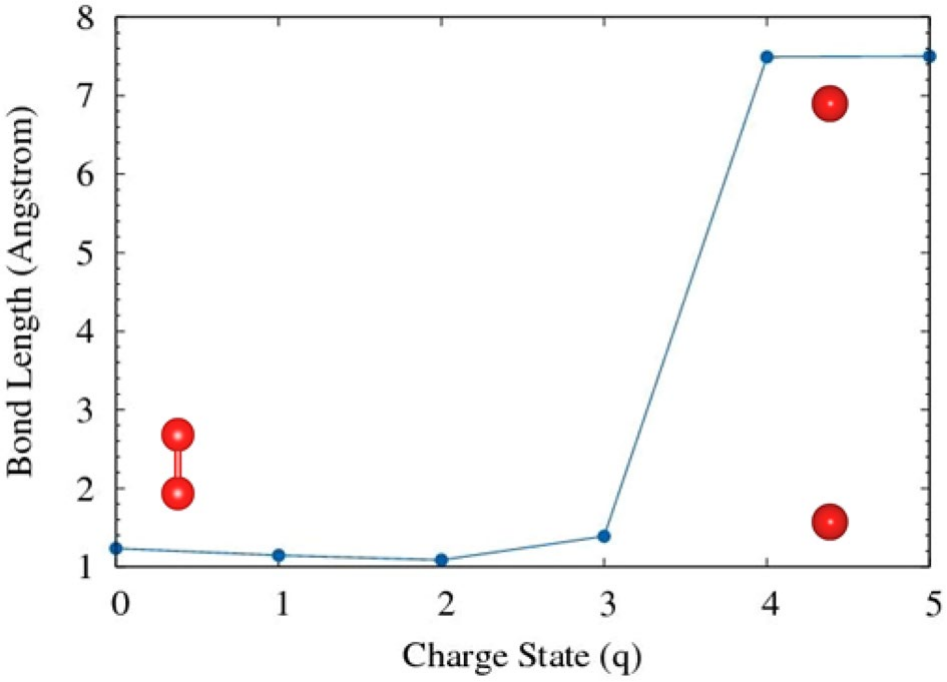
The effect of electron removal in oxygen molecule. The dots represents the O–O bond,the relative bond distance is indicated by O atoms.

First, the oxygen (O$_{2}$) molecule was optimized and bond distance and equilibrium energy were obtained. The O–O bond length was found to be 1.233 Å, which is consistent with experimental and previously reported computational values. In O$_{2}$ molecule, we notice that gradual electron extraction shows initially the bond length contracts slightly then expands for when a large number of electrons are removed (see Fig. 3). The bond length variation as a function of the charge state of an oxygen molecule is presented in Table  1. The removal of electrons weakens the bond strength and hence the bond-dissociation energy is reduced. In a pure oxygen molecule, the bond-dissociation energy is stronger due to the formation of double bonds (119 kcal/mole or 5.15 eV/bond).

Figure 3 shows the bond length as a function of the charge state (q). The x-axis label 1 refers to the + 1 charge state with removing one electron, resulting in a positive charge in the molecule. We observed that with q = + 4 (4 electrons are removed) demonstrates the dissociation of the bonds. The distance (d = 7.5 Å) is due to chosen box size of 15 Å, indicating that they are isolated from each other.

**Table 1:** The effect of electron removal in the bond length (d) of O$_{2}$ molecule.

Charge (q)	Bond length d (Å)	$\Delta$ d (Å)
0	1.233	0
+ 1	1.146	− 0.087
+ 2	1.085	− 0.148
+ 3	1.387	+ 0.154
+ 4	7.491	+ 6.258
+ 5	7.500	+ 6.267

The charge state (q) refers to the number of electron removed from the neutral system. Thus, q = 1 refers to 1.60217662 $\times 10^{-19}$ coulombs per molecule.

### Effect of electron extraction in water molecules

**Figure 4:**
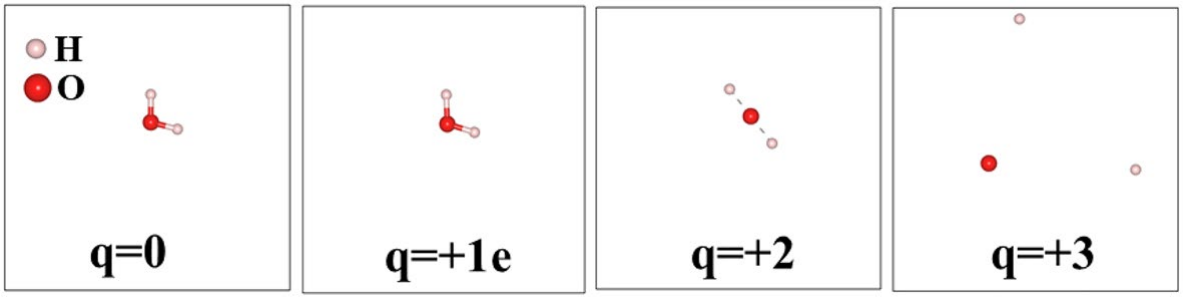
The effect of electron removal in water molecule.

**Figure 5:**
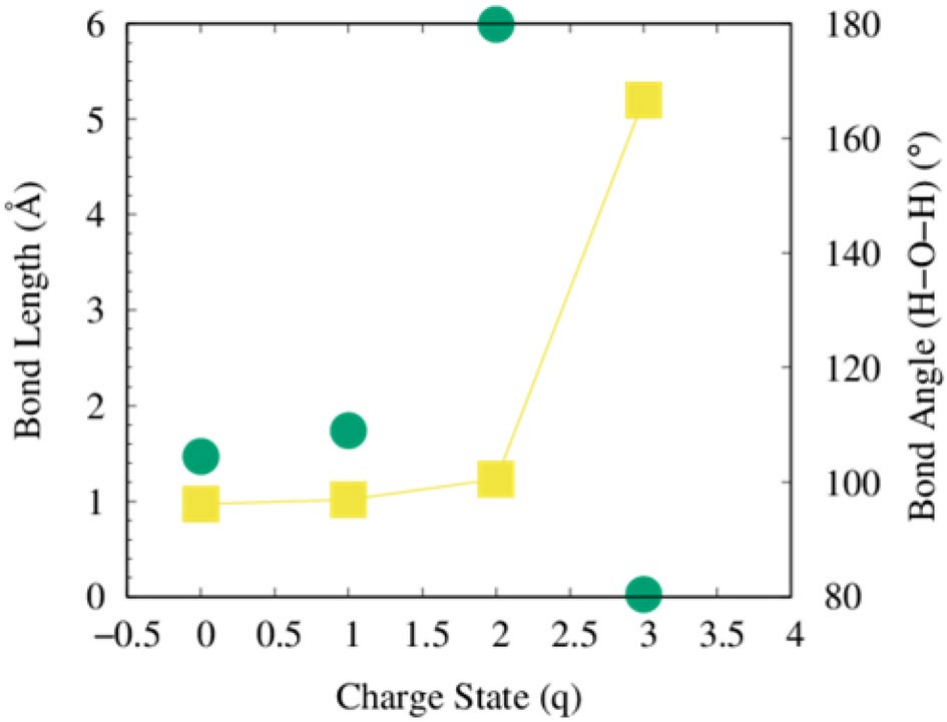
The effect of electron removal in water molecule. The dots represents the H–O–H bond angles.

Water molecules (H$_{2}$O) which are ubiquitous and are a significant part of life processes, stabilized as a tri-atomic molecule with *C2v* molecular symmetry and bond angle of 104.5$^{\circ }$  between the oxygen atom and the two hydrogen atoms. The H–O bond length is close to the bond (O–H) length of 0.9572 Å  and the bond angle (H–O–H) of 104.5 $^{\circ }$. Our calculated data are in very good agreement with the experimental reports, as shown in Table  2. We observed that upon extraction of electrons from the water molecule, both the bond lengths (H–OH) and the bond angle (H–O–H) change significantly as shown in Figs. 4 and  5. Note that these calculations have been performed in a vacuum. The pathway of dissociation of the water molecule, surrounded by other water molecules would be significantly different since in an aqueous environment formation of OH-radical is more likely. In Fig. 3, none of these scenarios correspond to the formation of OH-radical, simply because of geometrical symmetry of a single H$_2$O molecule and the periodic boundary condition used in the DFT calculation.

**Table 2:** The effect of electron removal in the bond length of H$_{2}$O molecule.

Charge (q)	Bond Length d (Å)	$\Delta$ d (Å)	$\Theta$ $^{\circ }$	$\Delta \Theta$ $^{\circ }$
0	0.972	0	104.502	0
+ 1	1.017	+ 0.045	109.004	+ 4.502
+ 2	1.232	+ 0.260	179.862	+ 75.360
+ 3	5.200	+ 4.228	80.416	− 24.086

The charge state (q) refers to the number of electron removed from the neutral system. Charge q = 1 refers to 1.60217662 $\times 10^{-19}$ coulombs per molecule. The H-O-H bond angle is also provided.

Dissociation of HO–H bond in a water molecule needs approximately 118.8 kcal/mol (497.1 kJ/mol) when there is no charge involved. The bond energy of the covalent O–H bonds of the water molecule is approximately 110.3 kcal/mol (461.5 kJ/mol)^35^. In the case of the ionizing environment, these values will be reduced and hence, the reduction causes the fragmentation of the bonds easily as shown in Fig. 4. Figure  5 shows how the bond length and the angles changes with increasing electron removal from the water molecule.

For a highly ionizing environment, the bond angles will deviate from the angular to planar before breaking the bonds as shown in the case of charge state (q = 2). This indicates that the 2 electrons extraction per water molecule (3.20435324 $\times 10^{-19}$ coulombs per molecule) is sufficient to drive the fragmentation into ions.

### Effect of electron extraction in guanine and in a fragment of DNA molecule

**Figure 6:**
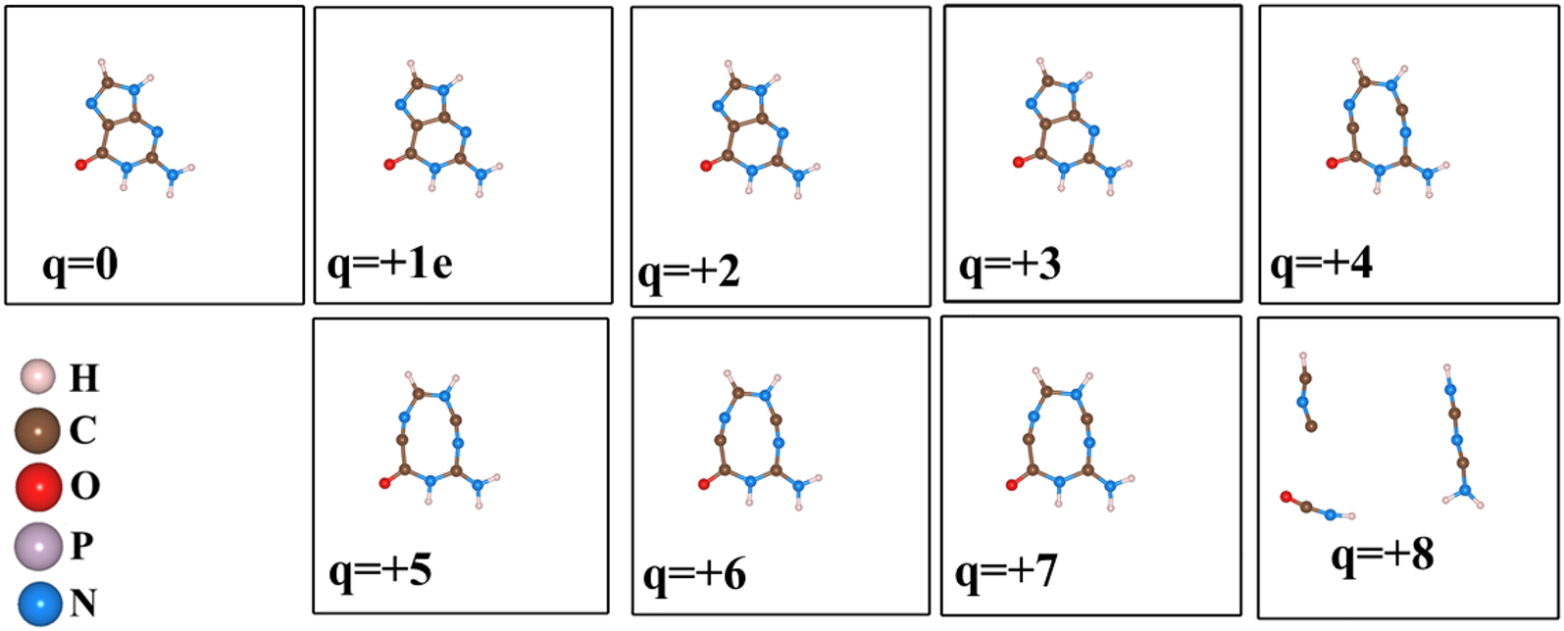
The effect of electron removal in Guanine molecule.

**Figure 7:**
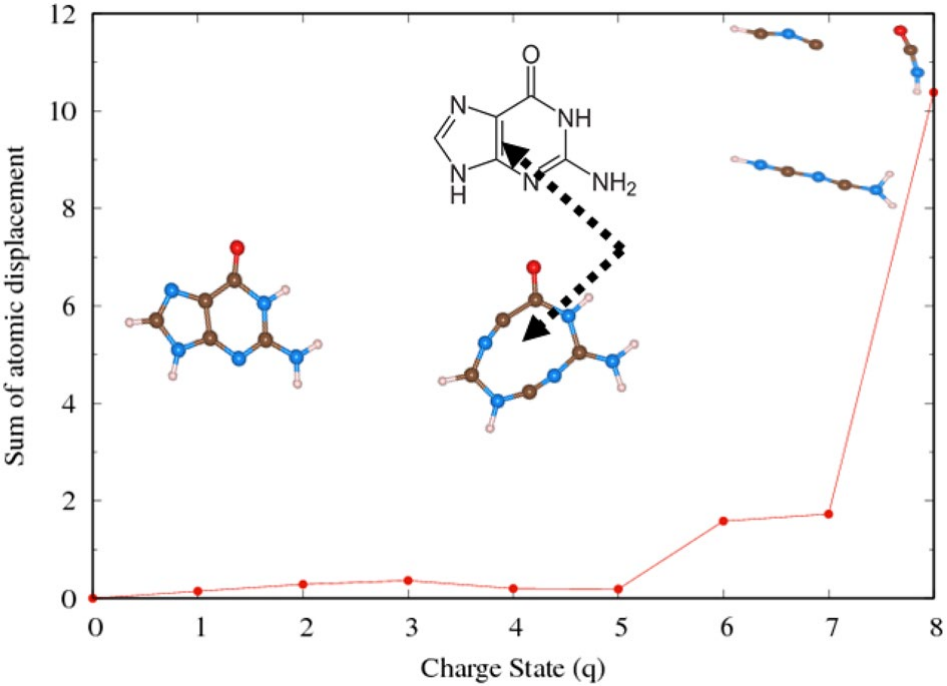
The sum of the displacement of the atoms as a function of charge (q) in a Guanine. The sum of the displacement is in Å and charge is in terms of number of electrons removed.

**Figure 8:**
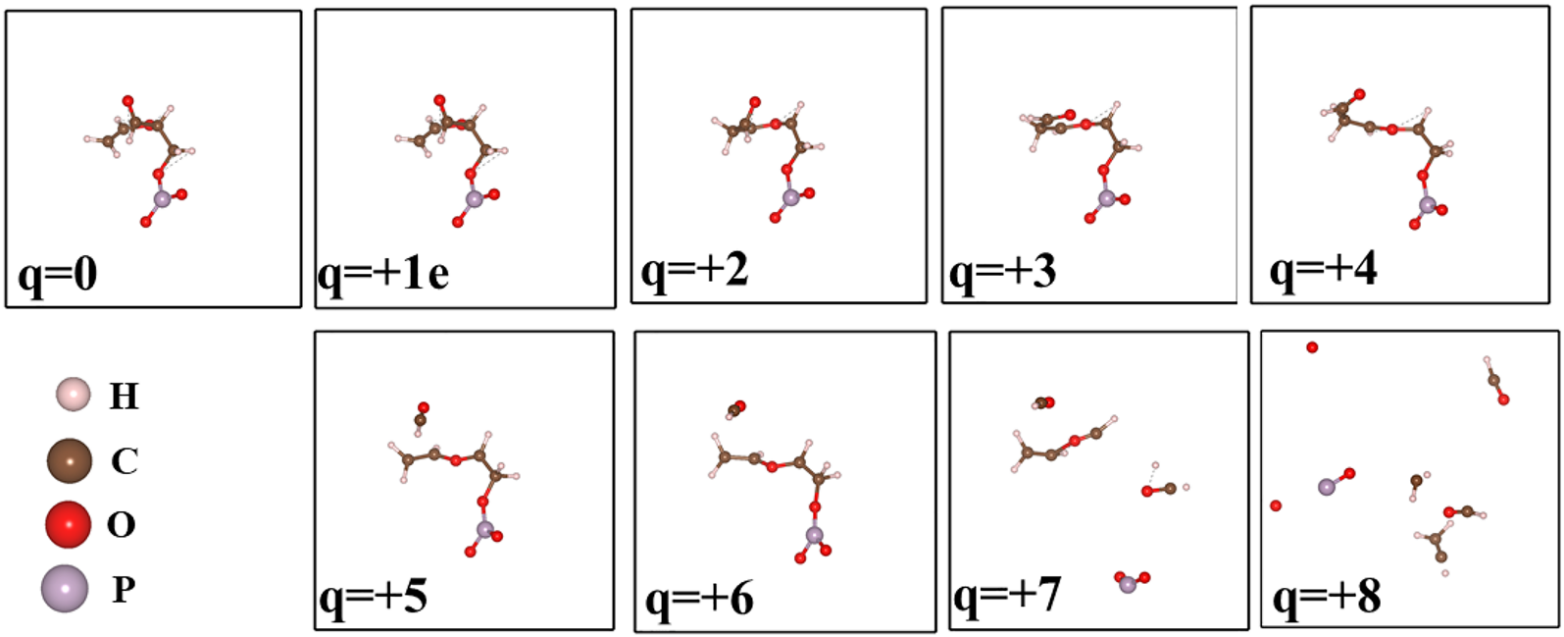
The effect of electron removal in a fragment of DNA molecule.

In this subsection, we present the impact of the electron extraction in guanine and a fragment of DNA molecule. Guanine, as shown in Fig. 6, is one of the four main nucleobases in DNA. It (2-amino-1,9-dihydro-6H-purin-6-one: IUPAC) consists of a fused pyrimidine-imidazole ring system with conjugated double bonds and has a planar molecular structure. To avoid spurious interaction due to the periodic boundary condition (PBC) in DFT calculation, a large simulation box was adopted for each molecule. Since, these molecules have multiple bonds, instead of monitoring individual bond length, we note the sum of the atomic displacements compared to the initial configurations. The gradual fragmentation of the Guanine molecule is observed as shown in Fig.  7. The corresponding sum of the displacements of the atoms as a function of charge states is shown in Fig.  8. We noticed that when the charge state is 4e, the C–C double bond is broken that will drive the structural instability.

Similarly, upon extraction of electrons from a fragment of DNA as shown in Fig. 8, bonds start to change and dissociate when the change is sufficient such as q = 4e. Eventually, the molecule starts to collapse into smaller fragments in a sufficiently high ionization environment. This indicates that these molecules are prone to damage when exposed to an ionizing radiation environment.

Ionizing radiation can extract electrons from these molecules resulting in ions that can trigger bond dissociation. Our results indicate that radiation directly affects DNA atomic structure by causing fragmentation. In addition, there might be secondary effects such as the creation of reactive oxygen species that oxidize proteins and lipids, and cause damages to DNA, eventually, the overall effect might cause cell death and mitotic catastrophe^36^.

**Figure 9:**
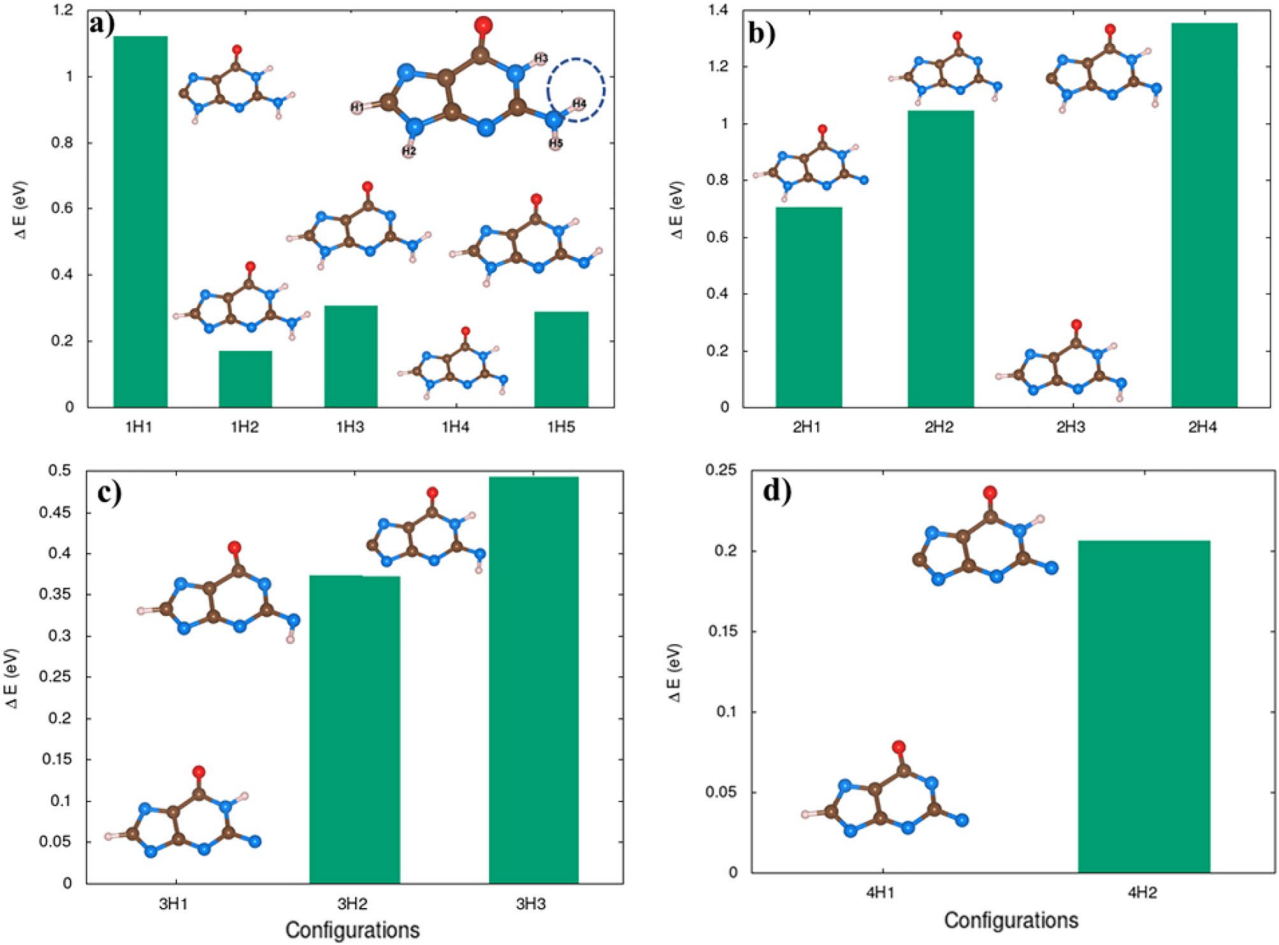
The effect of hydrogen removal in Guanine molecule. Notation—1H1 refers to one H removed the first configuration, 1H5 refers to 1H removed and is the fifth configuration. Similarly, 2H1: 2H atom removed and is the first configuration considered and so on. The energy of the configuration is with respect to the energetically most stable configuration in each H contents.

### Effect of hydrogen contents in the electron extraction and stability of DNA

Hydrogen deficient molecules, the fundamental DNA damage in the indirect process, were also investigated in order to check their dependence on the charge-induced dissociation. For illustration, the H atoms were gradually removed from Guanine as shown in Fig.  9. Interestingly, the fragmentation of the molecules occurs with less electron removal. Guanine cyclic ring is stable up to charge state of 3e if it had gone through three hydrogen atoms abstraction. The molecular geometry of guanine shows significant modification for 4 or 5H atoms abstraction. In 5H deficient case, the charge state of 3e completely dissociates the molecule into the molecular chain.

**Figure 10:**
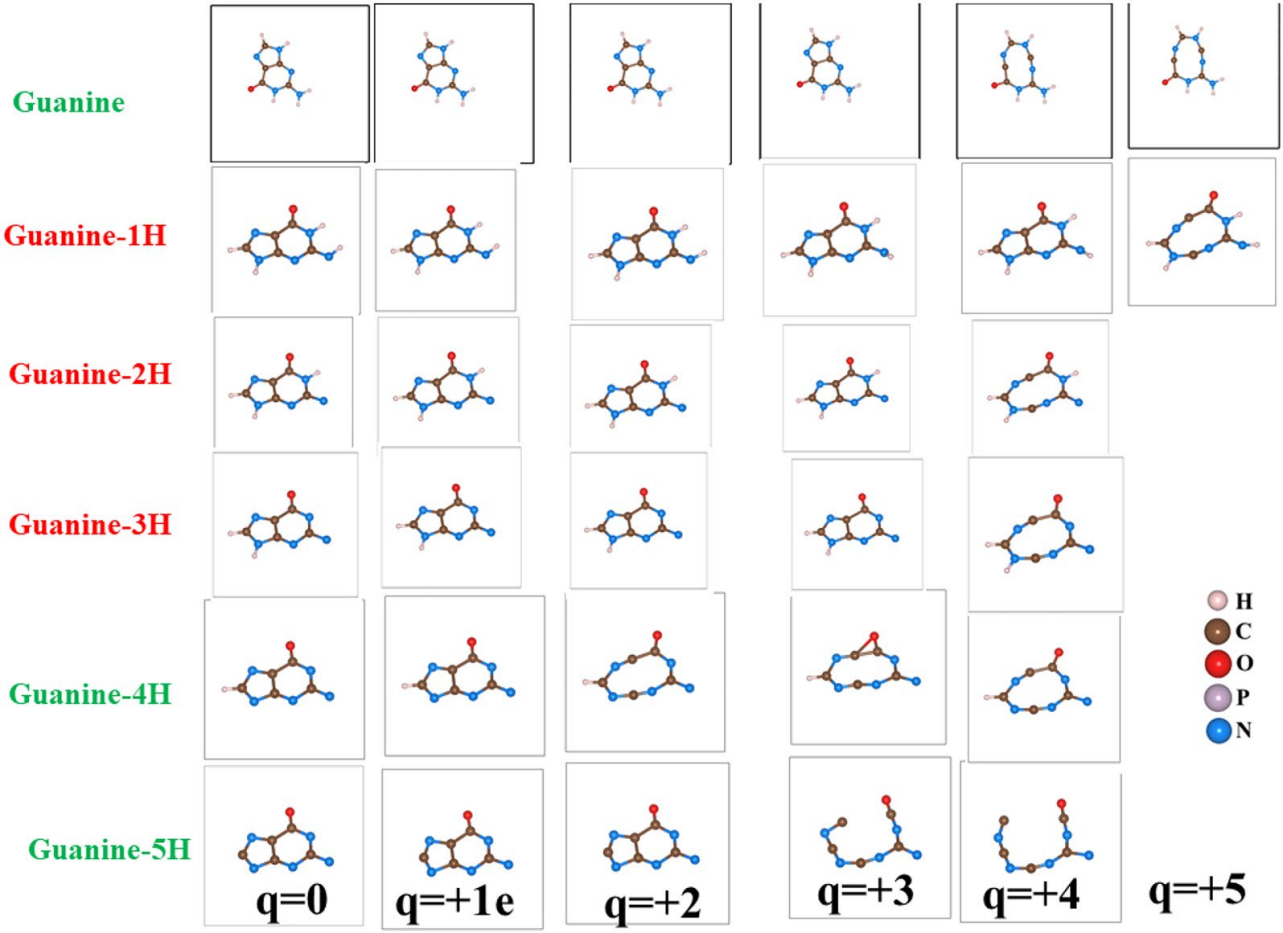
The effect of electron extraction in hydrogen reduced Guanine molecule.

According to our calculations, guanine does not dissociate only upon removal of H atoms ($q = 0$ in Fig. 10). The combined effect of charge and reduced H environment, as illustrated in Fig. 10 will lead to the fragmentation of these molecules. This indicates that the charge-induced dissociation of molecules strongly depends on the hydrogen environment. Note that reoptimizations of the molecular geometry of guanine after the removal of H atoms, have resulted in slightly different initial geometries. Different structural configurations as a function of number of hydrogen abstractions of guanine influence the stability of molecule due to subsequent removal of electrons. The optimized geometry has shown differently if we had removed the electrons first. Therefore, these two operations, hydrogen abstraction and electron-removal do not commute as their corresponding optimized molecular structure are not identical. This observation suggests implication of local structural change in the actual scenario of DNA damage as the H-abstraction is a much slower process compared to direct damage that is basically the electron removal.

Because the indirect damage is a slow process, due to reaction–diffusion of OH-radicals, we attribute the direct damage following by indirect damage to the interaction of DNA with a single track of radiation that is responsible for linear-term in the linear-quadratic cell survival model. The second scenario that is the removal of H and a subsequent direct ionization is most likely relevant to DNA damage induced by two tracks that is relevant to the quadratic term in the linear-quadratic cell survival model.

It is substantial to point out the current Monte Carlo (MC) codes utilized in studying the impact of radiation on biological materials lack the details since they use some empirical values for the excitations and DNA damage.^24,37^ Therefore, the present first-principles-based calculation provides important input parameters to take into account in those models.

## Conclusion

Using DFT calculations, we systematically investigated the atomic bond dissociation in an ionization environment and the fragmentation behavior of the DNA base pair molecule along with water and oxygen molecules. Our results demonstrate that the bond fragmentation is proportional to the charge of the molecule and there is the limitation of the charge density of the molecule that it can withstand before collapsing into fragments. This highlights the importance of using the optimal dose of radiation for safe use. Moreover, the bond dissociation behavior strongly depends on the hydrogen contents of the molecule. A hydrogen-reduced environment is detrimental to radiation-induced molecule fragmentation. This research is very applicable in radiation therapy as well as an environment where the human body will be exposed to radiation environments such as nuclear power plants or voyage to outer space. Thus, this study shed light on the atomic-level details of the mechanism of bond dissociation in the presence of ionizing radiation.

## Data Availability

Derived data supporting the findings of this study are available from the corresponding authors upon request.
